# 3D-Drawn Supports for Ion-Selective Electrodes

**DOI:** 10.1021/acs.analchem.1c05431

**Published:** 2022-02-17

**Authors:** Justyna Kalisz, Katarzyna Wȩgrzyn, Krzysztof Maksymiuk, Agata Michalska

**Affiliations:** Faculty of Chemistry, University of Warsaw, Pasteura 1, 02-093 Warsaw, Poland

## Abstract

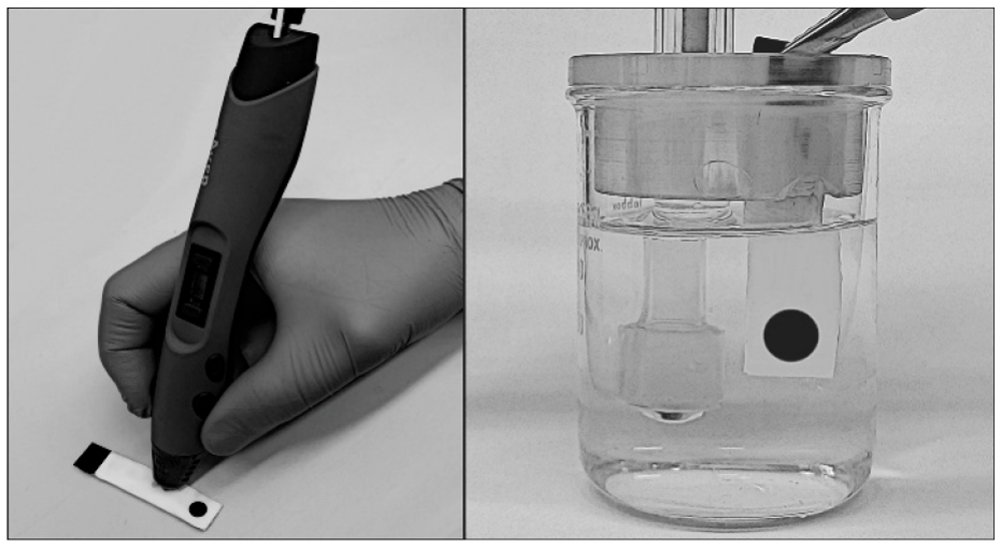

A new concept of easy to make, potentially disposable potentiometric sensors is presented. A thermoprocessable carbon black-loaded, electronically conducting, polylactide polymer composite was used to prepare substrate electrodes of user’s defined shape/arrangement applying a 3D pen in a hot melt process. Covering of the carbon black-loaded polylactide 3D-drawn substrate electrode with a PVC-based ion-selective membrane cocktail results in spontaneous formation of a zip-lock structure with a large contact area. Thus, obtained ion-selective electrodes offer sensors of excellent performance, including potential stability expressed by SD of the mean value of potential recorded equal to ±1.0 mV (*n* = 6) within one day and ±1.5 mV (*n* = 6) between five days. The approach offers also high device-to-device potential reproducibility: SD of mean value of *E*^0^ equal to ±1.5 mV (*n* = 5).

## Introduction

The concept of coated-wire potentiometric sensors presented nearly 50 years ago^1^ has had a pronounced effect on the field of ion-selective electrodes (ISEs). The major attractiveness (and the main drawback) of the coated-wire approach lays in the simplicity of the construction that brings together an electron conductor (metal wire or glassy carbon) and an ionic conductor: ion-selective membrane (ISM). This, however, results in a blocked interface at which activity of the spontaneously formed redox systems (metal oxides, water droplets) contributes to potential reading stability issues. To overcome this drawback introduction of a transducer layer in between metal and ISM was proposed.^2^ This idea has stimulated many researches ultimately leading to improved potentiometric sensors, novel applications, or alternative readout modes.^3−5^

Different systems have been proposed as transducer layers,^3,4^ among the most successful proposed are nanostructural carbon materials: carbon nanotubes,^6−8^ mesoporous carbon,^9,10^ or carbon black (CB).^11−13^ If the transducer forms either a separate layer or a composite with ISM, improved potential stability is observed,^12,14^ and analytical performance is comparable to that of classical ISEs with internal solutions.^3,6−14^

Usually the transducer layer needs to be applied on a macroscopic substrate electrode; thus, the sensors prepared are restricted to the form of the substrate electrode, being clearly a constrain at least for certain applications. For practical applications, for example, for disposable ISEs, planar, screen printed substrates were considered. Adhesion problems (leading to irreproducibility of recorded potentials) and hydrolysis of conductive paste components (resulting in formation of lipophilic compounds that can be spontaneously accumulated in ISM ultimately adversely affecting its analytical performance) are the main drawbacks of this approach.^15^ This becomes important especially because potentiometric applications (typically) require longer times compared to, for example, amperometric or voltammetric experiments. On the other hand, it should be stressed that applications of ISMs are usually done by drop casting of solvent-based mixtures of components—contact of the solvent with the screen printed substrate can also result in partial dissolution and ultimately in unwanted and uncontrolled change of the membrane composition.

Alternatively, the conducting track can be prepared by drop casting, spraying, and painting of conductive materials dispersion, followed by drying.^7,8,16−18^ This typically requires applications of aqueous suspensions containing stabilizing agents, for example, surfactants.^7,18^ In consequence, the tendency to accumulate water in the transducer phase and/or stabilizer release to the ISM is leading to potential stability issues, especially in longer time scales.^7^ Clearly, there is room for improvements as far as the choice of materials used to prepare disposable ISEs and the method of its application are concerned, especially if the requirements of reduced calibration (or calibration free) sensors should be met.

In this work, we propose a simple method allowing preparation of potentiometric sensors of high potential stabilities in time and device-to-device reproducibility using hot melt fabrication of electrode platforms. For the first time, thermoprocessable conductive material, for example, carbon black (CB), dispersed within a polymer matrix and a 3D manufacturing approach are used to prepare ISEs. Preparation of a conductive track/supporting electrode using thermoprocessable material eliminates dispersion agents or solvents from the sensor substrate preparation step. 3D printing of CB-loaded polymers was used to prepare electrochemical sensing platforms or substrate electrodes,^19,20^ and other composites were used to prepare reference electrodes^21^ but not ISEs. 3D printing requires a printer and software to design the object to be printed, making it potentially suitable for larger scale sensor production. In the laboratory scale or to meet the requirements of easy preparation, 3D pen, a low cost alternative for 3D printing, is attractive.^22,23^ To the best of our knowledge neither 3D printing nor 3D drawing, was considered previously in the context of preparation of ISEs.

From a materials point of view, this approach seems highly promising, too. 3D technologies often use polylactide (PLA), which to the best of our knowledge has not been considered as construction material for ISEs. To prepare a conductive layer PLA loaded with CB, a proven effective transducer material,^11−13^ can be used. Moreover, PLA is partially soluble in a THF membrane solvent, and it is known to form blends with PVC,^24^ the effect that is likely to improve adhesion of sensors layers, especially in the presence of the plasticizer used for ISM preparation. The important additional benefit is that 3D-drawn supporting electrodes can be easily obtained in any form/shape, even in low resources conditions—the limit is the imagination of the drawing person. The herein proposed novel concept was applied to prepare 3D-K^+^-ISEs, 3D-Ca^2+^-ISEs, and 3D-Cl^–^-ISEs as proofs of concept.

## Experimental Section

### Material and Reagents

Filament: (plain) PLA (ultrafuse white, 1.75 mm diameter) from BASF and conductive composite CB-loaded material (CB-PLA; Proto-pasta, 1.75 mm diameter) from ProtoPlant, Inc. (USA) were used as obtained. The CB-PLA material according to the producer specification is composed of >65% polyactide resin, <21.43% carbon black, <12.7% (unspecified) polymer (w/w), and the ratio of PLA to CB in the material is roughly 3:1 by weight.

Other reagents used are described in the SI.

### Fabrication of 3D-Drawn Electrodes

**Figure 1:**
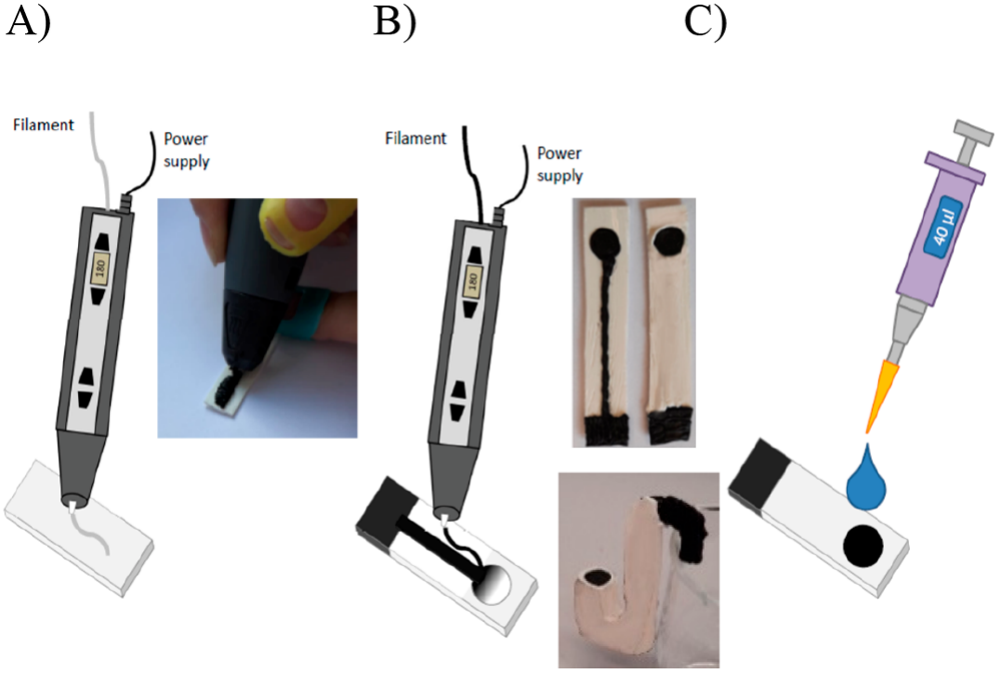
Schematic representation of sensors preparation: (A) insulating layer prepared using a PLA filament. (B) CB-PLA conductive track and support is drawn; pictures show the conductive track (left) and PLA-isolated conductive track leaving the exposed substrate. The substrate electrode can be of any shape including, for example, a “pipe type” electrode with the working surface facing up (lower picture). (C) Membrane is drop cast.

A 3D pen (Maker Factory, Germany) was used. The extrusion temperature was 180 °C both for PLA and CB-PLA. Various shapes of supporting electrodes were drawn (Figure S1); if not otherwise stated, the studies were conducted for a classical layout sensor with a circular supporting electrode. Crafting of the sensor is shown in Figure 1. On the top of the drawn frame (6 cm × 1 cm) of an insulating PLA back layer, a 2 mm wide conductive CB-PLA track (30 mm long) ending with a disk (0.8 cm diameter) was drawn, followed by applying insulation on the top, leaving a conductive opening of a 0.475 cm^2^ surface area. Thus, the obtained substrates were used for electrochemical testing of supporting electrodes. Alternatively, these were further modified by drop casting 50 μL (in three equal portions) of a classical PVC-based membrane cocktail in THF solvent into the opening of the supporting electrode (Figure 1). After evaporation of the membrane cocktail solvent, the sensors were conditioned overnight in a 10^–3^ M primary ion chloride solution. The estimated thickness of the membranes used (from the cross section of SEM image, Figure S1) was 120 ± 5 μm.

Potassium-selective electrode, 3D-K^+^-ISE, membranes contained (by weight) 1.4% sodium tetrakis[3,5-bis(trifluoromethyl)phenyl]borate (NaTFPB), 2.8% valinomycin, 63.7% bis(2-ethylhexyl)sebacate (DOS), and 32.1% poly(vinyl chloride) (PVC). A total of 100 mg was dissolved in 1 mL of tetrahydrofuran (THF). Alternatively, calcium- or chloride-selective sensors, 3D-Ca^2+^-ISE or 3D-Cl^–^-ISE, were prepared in the same manner, and details of the cocktail compositions are given in SI similar to details of the apparatus and procedures used.

## Results and Discussion

### Characterization of 3D-Drawn Support Electrodes and Their Interface with PVC-Based ISM

**Figure 2:**
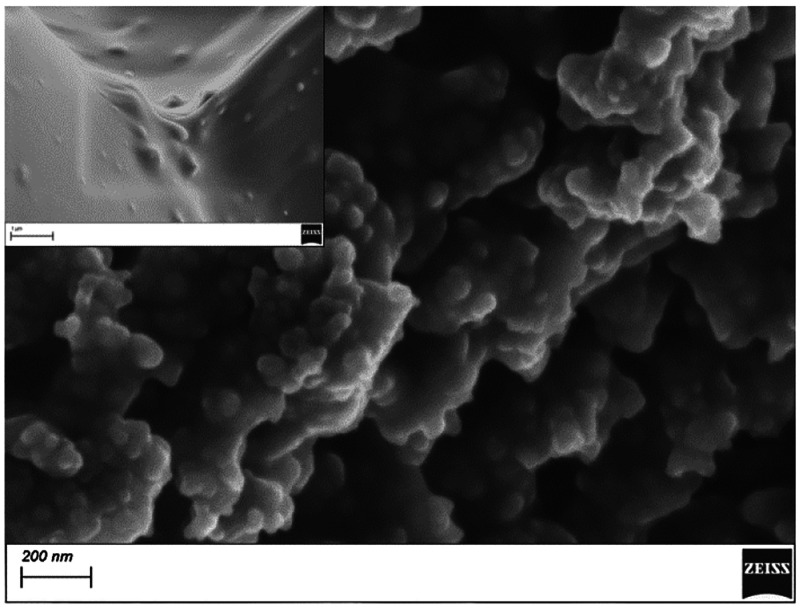
SEM images of surface of CB-PLA deposited film; inset is PLA.

In this work, thermoprocessable CB-PLA was used to prepare the conducting track and the support, whereas PLA was used to isolate a part of the track from contact with the solution to avoid using solvents or dispersion stabilizing agents.^7,8^ The morphologies of layers obtained from PLA and CB-PLA are significantly different (Figure 2).

The PLA surface is rather smooth, whereas CB-PLA is rough showing clear inclusions of fine (diameter <100 nm) spherical grains and agglomerates of CB partially embedded in PLA matrix. The increased contents of carbon in CB-PLA were confirmed using multipoint EDX analysis; details are given in Figure S2. The ratio of carbon to oxygen contents in CB-PLA was significantly higher and equal to 6.3:1, whereas for PLA, it was equal to 1.4:1.

It should be stressed that the effectiveness of (nonconductive) PLA isolation was confirmed by (anodic and cathodic) chronopotentiometric tests of the sensor working electrode area (Figure S3) performed in 0.1 M KCl solution. The change of current direction was accompanied by a potential drop resulting from the influence of ohmic resistance. Both for anodic and cathodic currents, a nonlinear dependence of potential vs time was obtained, pointing to a noncapacitive behavior of the studied material. The resistance values determined from the potential jump accompanying the different current direction (20.0, 13.0, and 6.0 kΩ for the surface areas of 0.23, 0.48, and 0.75 cm^2^, respectively) follow the change in the working electrode surface area, clearly confirming that PLA effectively isolates the CB-PLA layer from the solution (Figure S3A).

The substrates were also tested using electrochemical impedance spectroscopy within the frequency range of 0.01 Hz–100 kHz. The impedance spectra (complex plane impedance plots) are shown in Figure S3B. A relatively small semicircle in the high frequency range was recorded with an increasing diameter for a decreasing surface area. This diameter represents the ohmic resistance, and the obtained values are consistent with those obtained from chronopotentiometric experiments. In the range of lower frequencies, an almost linear dependence of −Z″ on Z′ was recorded, pointing to CPE behavior with a phase angle close to −80°. The CPE behavior suggests capacitive properties, and the deviation from −90° can result from some inhomogeneity (above-mentioned) of the surface. For the lowest frequencies, a small curvature for this dependence is visible, pointing to a highly inhibited charge transfer reaction occurring parallel to CPE.

Current–voltage characteristics of the CB-PLA substrate electrode, used without activation procedure,^19,20^ recorded at scan rates from 50 to 500 mV/s in 10 mM K_3_Fe(CN)_6_ in a 0.1 M KCl solution confirmed that the anodic/cathodic current peak is linearly dependent on square root of the scan rate, pointing to diffusion limitation (Figure S3C and D).

The CB-PLA and PLA layers obtained using 3D drawing were characterized with a similar water contact angle, equal to 60.8° ± 0.4 and 53.5° ± 0.3, respectively (Figure S4), pointing to relatively hydrophilic character.

**Figure 3:**
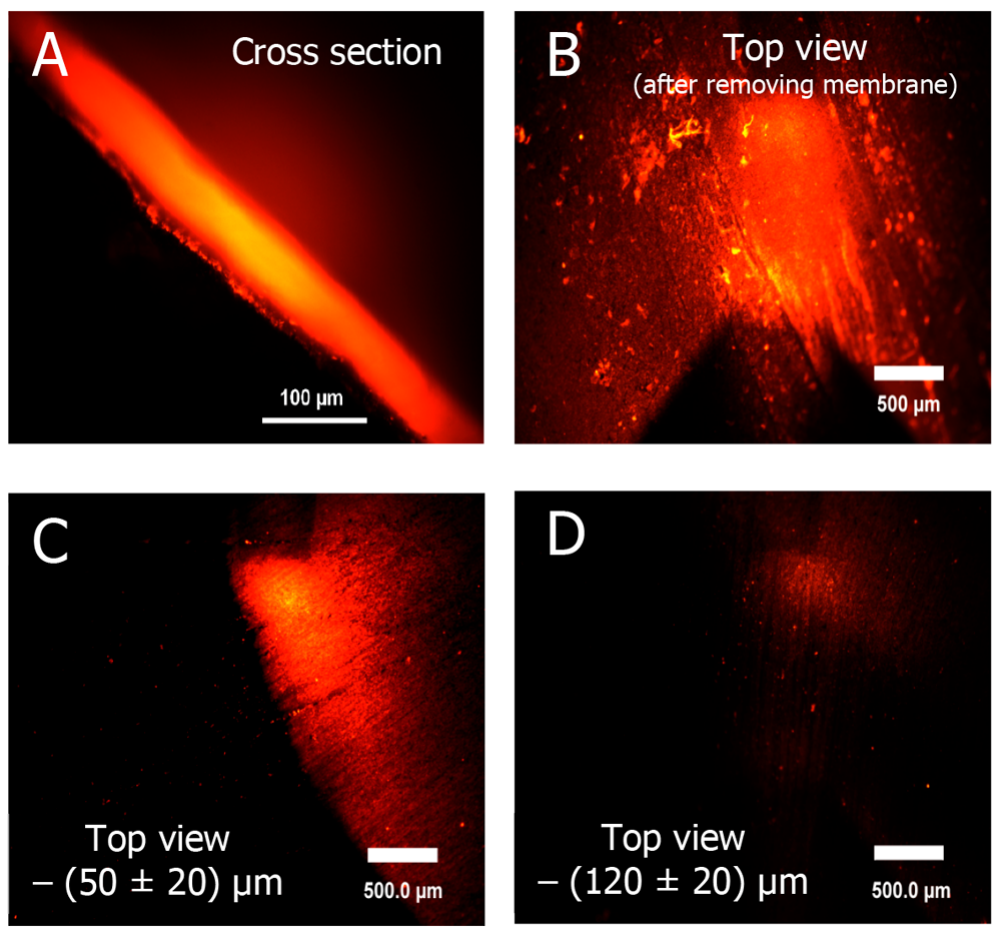
Fluorescence microscopy image of (A) cross section of dye containing ISM and CB-PLA. (B–D) Front views of CB-PLA surface post removing of ISM and after applying sand paper polishing.

To visualize formation of an intermediate layer between ISM and CB-PLA, a THF solution of Nile red (NR) dye was applied on the top of the conductive CB-PLA track, and the cross section of the system was studied using fluorescence microscopy (Figure S5). Figure S5 clearly shows that the dye penetrates the CB-PLA layer. The emission from the dye is observed from ca. 200 μm within CB-PLA. A similar effect was observed when a THF dispersed membrane, containing Nile Red, was applied on the top of CB-PLA (Figure 3). The cross-section image clearly shows dye emission from the membrane and CB-PLA layer, beneath the membrane. The CB-PLA surface, after removing of the membrane, clearly shows emission from the dye. Images taken after polishing—removing ca. 50 ± 20 μm and then 120 ± 20 μm of CB-PLA—also show emission of the dye, however, of smaller intensity. These experiments confirm that at the interface of ISM and PLA spontaneous mixing of components occurs, especially as both PVC and PLA can be plasticized by the ISM plasticizer. This effect is an important benefit of the herein proposed system. A spontaneously formed mixed layer, in the presence of CB, assures a large surface area and mechanical stability of the interface.

The above conclusions were confirmed by SEM (Figure S1C). The ISM and CB-PLA layers are clearly seen in the cross section, and the interface is rough. Cavities in the CB-PLA layer (shown in Figure 2 inset) are filled with ISM, ultimately forming a zip-lock structure. This is as expected for two immiscible polymers able to form blends.^24^ It should be stressed that on the contrary to other materials used to prepare tracks and supports for miniaturized disposable potentiometric sensors,^7^ CB-PLA is a clearly less water-absorbing material, characterized with a water diffusion coefficient comparable with that of plasticized PVC;^25^ thus, the risk of accumulation of water in the sensor is minimized.

### Electrochemical Responses of 3D-Drawn Support ISEs

**Figure 4:**
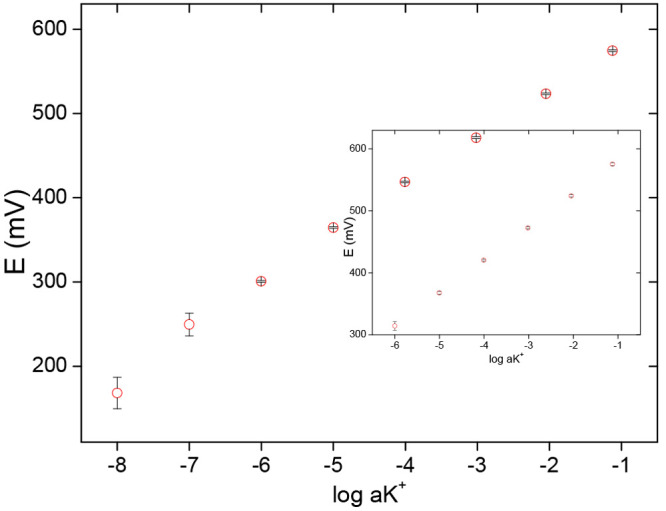
Potentiometric responses of 3D-K^+^-ISE electrode (within one day reproductibility) error bars show ± SD for *n* = 6 calibrations performed during one day. Inset: between days reproductibility error bars show ± SD for *n* = 6 calibrations performed during five days.

It seems rational to assume that the sensor prepared using the 3D support will be rather considered as a disposable one, yet both within day and between days reproducibility was tested. For K^+^-selective ISE prepared using 3D-drawn supporting electrodes, the dependence of the potential on a logarithm of activity of KCl was linear within the activity range from 10^–1^ to 10^–7^ M with the slope close to Nernstian and equal to 55.5 ± 0.6 mV (R^2^ = 0.999) (Figure 4). The potential reading reproducibility expressed as ± SD values for *n* = 6 calibrations performed during one day for concentrations from 10^–1^ to 10^–6^ M was close to 1.0 mV (Figure 4). The highly competitive reproducibility was for the first time achieved for craft-made sensors prepared from commercial, relatively inexpensive materials. Thus, the herein proposed approach is potentially attractive for limited resources/“on-demand” ISEs preparation.

The detection limits of herein proposed sensors were close to 10^–8^ M, and as observed previously^26^ for close to the detection limit KCl concentration (10^–7^ and 10^–8^ M), potential reading reproducibility expressed as SD was higher.

The between days potential reproducibility, *n* = 6 (five days) calibrations, is shown in Figure 4, inset. The SD of the mean potential value obtained for a given concentration is close to 1.5 mV, with the exception of the 10^–6^ M solution for which SD was equal to 6.8 mV. In our opinion, this is excellent reproducibility of the potential for a nominally disposable, craft-made, sensor in longer time scale. It should be stressed that 3D-K^+^-ISEs (made in one bath) were characterized with the standard potential scatter represented by SD of a mean value equal to ±1.5 mV for *n* = 5 handmade sensors. Thus, the device-to-device reproducibility is highly satisfactory acknowledging that sensors were handmade; i.e., differences can potentially result from, for example, slight deviations of the size of the substrate electrode or minor differences in the membrane thickness.

The results of chronopotentiometric and electrochemical impedance tests of 3D-drawn substrate ISEs are shown in Figure S6. In the chronopotentiometric experiment, as expected for high potential reading stability, a change of the potential recorded was in the range of a single mV either when cathodic or anodic current was applied. A linear dependence of potential vs time was recorded, both for anodic and cathodic currents, pointing to apparent capacitive behavior in the low frequency range. Using the method of Bobacka,^27^ the capacitance and resistance values were calculated from the recorded chronopotentiometric curves, and they were equal to 78 μF and 0.9 MΩ, respectively. The obtained capacitance value is higher than typically observed for a coated wire arrangement but significantly smaller than for typical solid-state ISEs. As expected, the resistance is much higher for 3D-K^+^-ISE compared to uncovered support and is consistent with the typical resistance of a PVC-based membrane.

In the impedance spectrum (Figure S6), two semicircles overlap to some extent, suggesting the presence of an inhibiting step in the charge transfer reaction. The resistance determined from the diameter of the semicircle recorded for higher frequencies is close to 0.6 MΩ, being around 2/3 of the resistance determined from the chronopotentiometric curve. This difference is typical for ISEs and was observed also for other systems. The higher resistance obtained under conditions of the chronopotentiometric experiment can be explained by concentration polarization in the membrane due to unidirectional current flow (in contrast to the EIS method where oscillations of the current around the equilibrium state occur). In the low frequency range, a linear dependence of −Z″ on Z′ was recorded with the slope close to 45°, suggesting the influence of Warburg impedance, due to diffusional limitations in the membrane.

Last but not the least, the results of the water layer test prove the lack of potential changes in time, confirming high stability of proposed sensors, with the exception of a small potential increase observed in interfering ion solutions (Figure S7). This effect can be attributed to a local increase of analyte concentration in the stagnant layer next to the ISM.

The selectivities of obtained 3D-K^+^-ISEs were not affected compared to other sensor constructions.^6,12^ The values of the logarithm of selectivity coefficients obtained as log *K*_pot K,J_ ± SD were equal to −3.7 ± 0.6, −3.6 ± 0.5, −3.9 ± 0.1, and −6.0 ± 0.2 for calcium, magnesium, sodium, and hydrogen ions, respectively.

Clearly, the herein proposed approach is not limited to 3D-K^+^-ISEs. Similar sensors, calcium- and chloride-ISEs, were also tested. 3D-Ca^2+^-ISE offered a slope equal to 24.6 ± 1.2 mV/dec (R^2^ = 0.995), and the detection limit equal to 10^–4.6^ M within the range from 10^–1^ to 10^–4^ M and within one day reproducibility of potential values recorded was lower than 1.5 mV (for *n* = 5 calibrations) (Figure S8). For 3D-Cl^–^-ISE within the range from 10^–1^ to 10^–5^ M, the slope equal to −52.2 ± 0.3 mV/dec (R^2^ = 0.999) and detection limit equal to 10^–5.2^ M were obtained. The within one day reproducibility of potential values recorded was somewhat worse compared to cation ISEs, and it was close to 2.5 mV (*n* = 4 calibrations) for the linear response range (Figure S9).

## Conclusions

Herein, we proposed a new type of ISE, easy to make, potentially disposable, and allowing preparation of sensors of any shape. The preparation of sensor substrates does not require a specialized setup or conditions allowing preparation under limited resource conditions. The proposed sensor platform hot melt preparation method does not require application of solution/paste processable (organic solvent dispersible) materials. Therefore, this approach is highly suitable for applications together with ion-selective polymeric membranes, eliminating the risk of uncontrolled transfer of the substrate/transducer material to the receptor phase. Moreover, ISEs prepared using 3D-drawn substrates offer outstanding performances, including high potential readings reproducibility and device-to-device reproducibility, despite the “craft” method of sensor preparation. The herein proposed novel approach can be easily extended to be applicable for other ion-selective sensor application modes.

## Supporting Information Available

Experimental details, pictures of different 3D-drawn supporting electrodes, SEM image of cross section of CB-PLA-ISM interface, EDX analysis results of PLA and CB-PLA, results of electrochemical tests of 3D-drawn substrate electrodes, water contact angles of PLA and CB-PLA, fluorescence microscopy image of the cross section of CB-PLA modified with Nile red, results of electrochemical studies and water layer test of 3D-K^+^-ISE, and potentiometric responses of calcium and chloride selective sensors. (PDF)

## References

[ref1] Cattrall R. W.; Freiser H. Coated Wire Ion-Selective Electrodes. Anal. Chem. 1971, 43, 1905–1906. 10.1021/ac60307a032. 22309557

[ref2] Buck R. P. Ion Selective Electrodes. Anal. Chem. 1976, 48, 23R–39R. 10.1021/ac60369a004. 22401014

[ref3] Hu J.; Stein A.; Buhlmann P. Rational Design of All-Solid-State Ion-Selective Electrodes and Reference. Electrodes TrAC 2016, 76, 102–114. 10.1016/j.trac.2015.11.004.

[ref4] Shao Y.; Ying Y.; Ping J. Recent Advances in Solid-Contact Ion-Selective Electrodes: Functional Materials, Transduction Mechanisms, and Development Trends. Chem. Soc. Rev. 2020, 49, 4405–4465. 10.1039/C9CS00587K. 32458836

[ref5] Bakker E. Enhancing Ion-Selective Polymeric Membrane Electrodes by Instrumental Control. TrAC 2014, 53, 98–105. 10.1016/j.trac.2013.09.014.

[ref6] Crespo G. A.; Macho S.; Rius F. X. Ion-Selective Electrodes Using Carbon Nanotubes as Ion-to-Electron Transducers. Anal. Chem. 2008, 80, 1316–1322. 10.1021/ac071156l. 18271511

[ref7] Jaworska E.; Maksymiuk K.; Michalska A. Optimizing carbon nanotubes dispersing agents from the point of view of ion-selective membrane based sensors performance - introducing carboxymethylcellulose as dispersing agent for carbon nanotubes based solid contacts. Electroanalysis 2016, 28, 947–953. 10.1002/elan.201500609.

[ref8] Kałuża D.; Jaworska E.; Mazur M.; Maksymiuk K.; Michalska A. Multiwalled Carbon Nanotubes-Poly(3-octylthiophene-2,5-diyl) Nanocomposite Transducer for Ion-Selective Electrodes: Raman Spectroscopy Insight into the Transducer/Membrane Interface. Anal. Chem. 2019, 91, 9010–9017. 10.1021/acs.analchem.9b01286. 31199120

[ref9] Lai C.-Z.; Fierke M. A.; Stein A.; Buhlmann P. Ion-Selective Electrodes with Three-Dimensionally Ordered Macroporous Carbon as the Solid Contact. Anal. Chem. 2007, 79, 4621–4626. 10.1021/ac070132b. 17508716

[ref10] Hu J.; Zou X. U.; Stein A.; Buhlmann P. Ion-Selective Electrodes with Colloid-Imprinted Mesoporous Carbon as Solid Contact. Anal. Chem. 2014, 86, 7111–7118. 10.1021/ac501633r. 24983327

[ref11] Paczosa-Bator B. Effects of Type of Nanosized Carbon Black on the Performance of an All-Solid-State Potentiometric Electrode for Nitrate. Microchim. Acta 2014, 181, 1093–1099. 10.1007/s00604-014-1216-7.

[ref12] Paczosa-Bator B. All-Solid-State Selective Electrodes Using Carbon Black. Talanta 2012, 93, 424–427. 10.1016/j.talanta.2012.02.013. 22483932

[ref13] Paczosa-Bator B. Ion-Selective Electrodes with Superhydrophobic Polymer/Carbon Nanocomposites as Solid Contact. Carbon 2015, 95, 879–887. 10.1016/j.carbon.2015.09.006.

[ref14] Stelmach E.; Maksymiuk K.; Michalska A. Dual Sensitivity Potentiometric and Fluorimetric Ion-Selective Membranes. Anal. Chem. 2021, 93 (44), 14737–14742. 10.1021/acs.analchem.1c03193. 34699175 PMC8581967

[ref15] Zielinska R.; Mulik E.; Michalska A.; Achmatowicz S.; Maj-Zurawska M. All-Solid-State Planar Miniature Ion-Selective Chloride Electrode. Anal. Chim. Acta 2002, 451, 243–249. 10.1016/S0003-2670(01)01407-6.

[ref16] Rostampour M.; Bailey B.; Autrey C.; Ferrer K.; Vantoorenburg B.; Patel P. K.; Calvo-Marzal P.; Chumbimuni-Torres K. Y. Single-Step Integration of Poly(3-Octylthiophene) and Single-Walled Carbon Nanotubes for Highly Reproducible Paper-Based Ion-Selective Electrodes. Anal. Chem. 2021, 93, 1271–1276. 10.1021/acs.analchem.0c04506. 33372767

[ref17] Jaworska E.; Pomarico G.; Berna B. B.; Maksymiuk K.; Paolesse R.; Michalska A. All-Solid-State Paper Based Potentiometric Potassium Sensors Containing Cobalt(II) Porphyrin/Cobalt(III) Corrole in the Transducer Layer. Sens. Actuators B Chem. 2018, 277, 306–311. 10.1016/j.snb.2018.08.090.

[ref18] Mensah S. T.; Gonzalez Y.; Calvo-Marzal P.; Chumbimuni-Torres K. Y. Nanomolar Detection Limits of Cd2+, Ag+, and K+ Using Paper-Strip Ion-Selective Electrodes. Anal. Chem. 2014, 86 (15), 7269–7273. 10.1021/ac501470p. 25023061

[ref19] Richter E. M.; Rocha D. P.; Cardoso R. M.; Keefe E. M.; Foster C. W.; Munoz R. A. A.; Banks C. E. Complete Additively Manufactured (3D-Printed) Electrochemical Sensing Platform. Anal. Chem. 2019, 91, 12844–12851. 10.1021/acs.analchem.9b02573. 31535844

[ref20] Cardoso R. M.; Mendonça D. M. H.; Silva W. P.; Silva M. N.T.; Nossol E.; da Silva R. A. B.; Richter E. M.; Muñoz R. A. A. 3D Printing for Electroanalysis: From Multiuse Electrochemical Cells to Sensors. Anal. Chim. Acta 2018, 1033, 49–57. 10.1016/j.aca.2018.06.021. 30172331

[ref21] Lewenstam A.; Bartoszewicz B.; Migdalski J.; Kochan A. Solid Contact Reference Electrode with a PVC-Based Composite Electroactive Element Fabricated by 3D Printing. Electrochem. Commun. 2019, 109, 106613 10.1016/j.elecom.2019.106613.

[ref22] de Oliveira F. M.; de Melo E. I.; da Silva R. A. B. 3D Pen: A Low-Cost and Portable Tool for Manufacture of 3D-Printed Sensors. Sens. Actuators B Chem. 2020, 321, 128528 10.1016/j.snb.2020.128528.

[ref23] Cardoso R. M.; Castro S. V. F.; Stefano J. S.; Muñoz R. A. A. Drawing Electrochemical Sensors Using a 3D Printing Pen. J. Braz. Chem. Soc. 2020, 31, 1764–1770. 10.21577/0103-5053.20200129.

[ref24] Hachemi R.; Belhaneche-Bensemra N.; Massardier V. Elaboration and Characterization of Bioblends Based on PVC/PLA. J. Appl. Polym. Sci. 2014, 131, 40045 10.1002/app.40045.

[ref25] Siparsky G. L.; Voorhees K. J.; Dorgan J. R.; Schilling K. J. Water Transport in Polylactic acid (PLA), PLA/ Polycaprolactone Copolymers, and PLA/Polyethylene Glycol Blends. J. Environ. Polym. Degrad. 1997, 5, 125–136. 10.1007/BF02763656.

[ref26] Michalska A.; Wojciechowski M.; Bulska E.; Maksymiuk K. Experimental Study on Stability of Different Solid Contact Arrangements of Ion-Selective Electrodes. Talanta 2010, 82, 151–157. 10.1016/j.talanta.2010.04.012. 20685450

[ref27] Bobacka J. Potential Stability of All-Solid-State Ion-Selective Electrodes Using Conducting Polymers as Ion-to-Electron Transducers. Anal. Chem. 1999, 71, 4932–4937. 10.1021/ac990497z. 21662838

