# Supplementary material for: 3D-Drawn Supports for Ion-Selective Electrodes
Source: Anal Chem. 2022 Feb 17;94(8):3436–40. doi: 10.1021/acs.analchem.1c05431 (PMC8892439; doi:10.1021/acs.analchem.1c05431)
Supplement: Supplementary file 1 — ac1c05431_si_001.pdf [file ac1c05431_si_001.pdf]

Supporting Information

3D-drawn supports for ion-selective electrodes

Justyna Kalisz†, Katarzyna Węgrzyn†, Krzysztof Maksymiuk, Agata Michalska*

Faculty of Chemistry, University of Warsaw, Pasteura 1, 02-093 Warsaw, Poland
* agatam@chem.uw.edu.pl, Phone:+48 22 55 26 331

Table of contents

1. Experimental details.

2. Pictures of different 3D-drawn supporting electrodes and SEM image of cross-section of CB-PLA-ISM interface.

3. EDX analysis results of PLA and CB-PLA.

4. Results of electrochemical tests of 3D-drawn substrate electrodes.

5. Water contact angle of PLA and CB-PLA.

6. Fluorescence microscopy image of the cross section of CB-PLA modified with Nile red.

7. Results of electrochemical studies and water layer test of 3D-K^+-ISE

8. Potentiometric responses of calcium selective sensors.

9. Potentiometric responses of chloride selective sensors.

Experimental

Chemicals

Valinomycin, calcium ionophore IV (ETH5234), sodium tetrakis[3,5-bis-(trifluoromethyl)phenyl]borate (NaTFPB), tridodecylmethylammonium chloride (TDMAC), bis(2-ethylhexyl)sebacate (DOS), 2-nitrophenyl octyl ether (oNPOE), poly(vinyl chloride) (PVC), and tetrahydrofuran (THF) were obtained from Sigma Aldrich. Used salts were of analytical grade and were obtained from POCh (Gliwice, Poland). Ultrapure water with a resistivity of 18.2 MΩcm (Milli-Q plus, Millipore) was used throughout the work.

Composition of calcium and chloride membranes used

Calcium selective electrodes, 3D-Ca^{2+}-ISE, membranes contained (by weight): 0.7% NaTFPB; 1% calcium ionophore IV; 67.9% oNPOE and 30.4% PVC. Chloride selective electrodes, 3D-Cl^{-}-ISE, membranes contained (by weight): 5% TDMAC, 62.0% DOS and 33.0% PVC. In all cases total 100 mg were dissolved in 1ml of THF.

Electrodes were prepared using the same procedure as for 3D-K^{+}-ISE.

Nile red solution and membrane

2 mg Nile red was dissolved in 1 ml of THF, alternatively 2 mg Nile red, 32.8 mg PVC and 65.2 mg DOS were dissolved in 1 ml THF.

Apparatus and techniques

In the potentiometric experiments, a multichannel data acquisition setup and software, Lawson Laboratories. Inc. (3217 Phoenixville Pike, Malvern, PA) was applied. The pump systems 700 Dosino and 711 Liquino (Metrohm, Herisau, Switzerland) were used to obtain sequential dilutions of calibrating solutions. The double junction Ag/AgCl reference electrode with 1 M lithium acetate in the outer sleeve (Möller Glasbläserei, Zürich, Switzerland) was used. The recorded potential values were corrected for the liquid junction potential calculated according to the Henderson approximation. Mean ion activities were calculated according to Debye−Hückel theory. Selectivity coefficients were determined using separate solution method within the concentration range from 10^{-1} to 10^{-4} M, in calculations the experimental slope values were used.

All electrochemical measurements were carried out using a galvanostat-potentiostat CH-Instruments model 760 A (Austin, TX, USA) in a three-electrode system. A conventional

electrochemical cell composed of Ag/AgCl electrode (3M KCl) as the reference electrode, a platinum wire as the counter electrode and the 3D-drawn PLA-ISE as the working electrodes was used. Cyclic voltammetry and electrochemical impedance spectroscopy (EIS) techniques were used for electrochemical characterization of the CB-PLA electrodes.

The morphological and structural characterization of 3D-drawn substrate electrodes was performed by using a LEO 435 VP model, Carl Zeiss microscope equipped with SE2 type detector.

Contact angle measurements were performed applying 1.5 μL droplet of water on a top of PLA using KSV Contact Angle Measurement System (Biolin Scientific). Contact angle values were determined as the mean of 3 measurements with an appropriate time interval collection. The contact angles were measured at room temperature on the left and right sides of the droplets.

Microscopic measurements were performed with a Nikon Eclipse LV 100 optical microscope operating in fluorescence or white light (reflection) mode.

A) B) C)

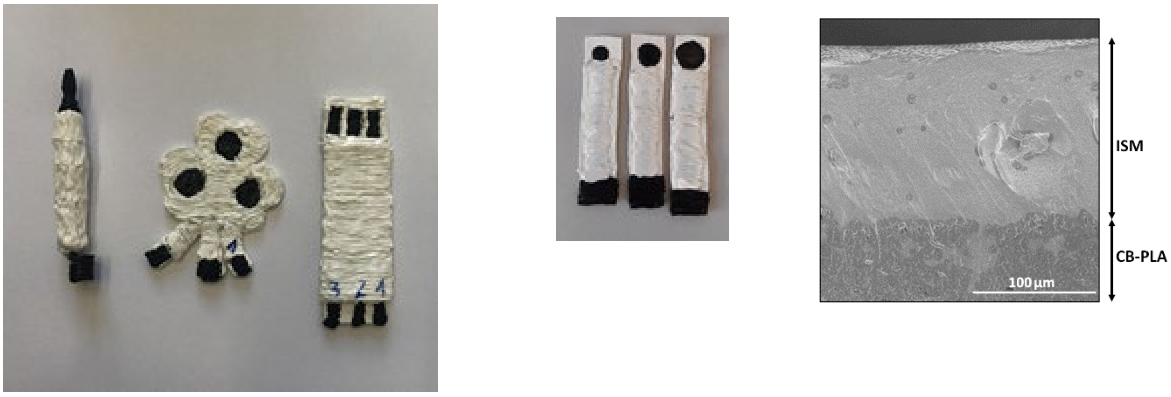

Figure S1. 3D-drawn supporting electrodes: A) various shapes of 3D-drawn supporting electrodes and electrodes sets prepared, B) changing area of working electrode. C) SEM image of cross-section of CB-PLA-ISM interface.

A)

Spectrum: Acquisition

Element	Series	unn. C [wt.%]	norm. C [wt.%]	Atom. C [at.%]	Error (1 Sigma) [wt.%]
Carbon	K-series	50.48	50.48	57.81	7.98
Oxygen	K-series	48.43	48.43	41.64	8.33
Aluminium	K-series	1.09	1.09	0.56	0.12
	Total:	100.00	100.00	100.00	

B)

Spectrum: Acquisition

Element	Series	unn. C [wt.%]	norm. C [wt.%]	Atom. C [at.%]	Error (1 Sigma) [wt.%]
Carbon	K-series	82.64	82.64	86.38	9.83
Oxygen	K-series	17.36	17.36	13.62	3.00
	Total:	100.00	100.00	100.00	

Figure S2. Copy of EDX analysis results of A) PLA and B) CB-PLA.

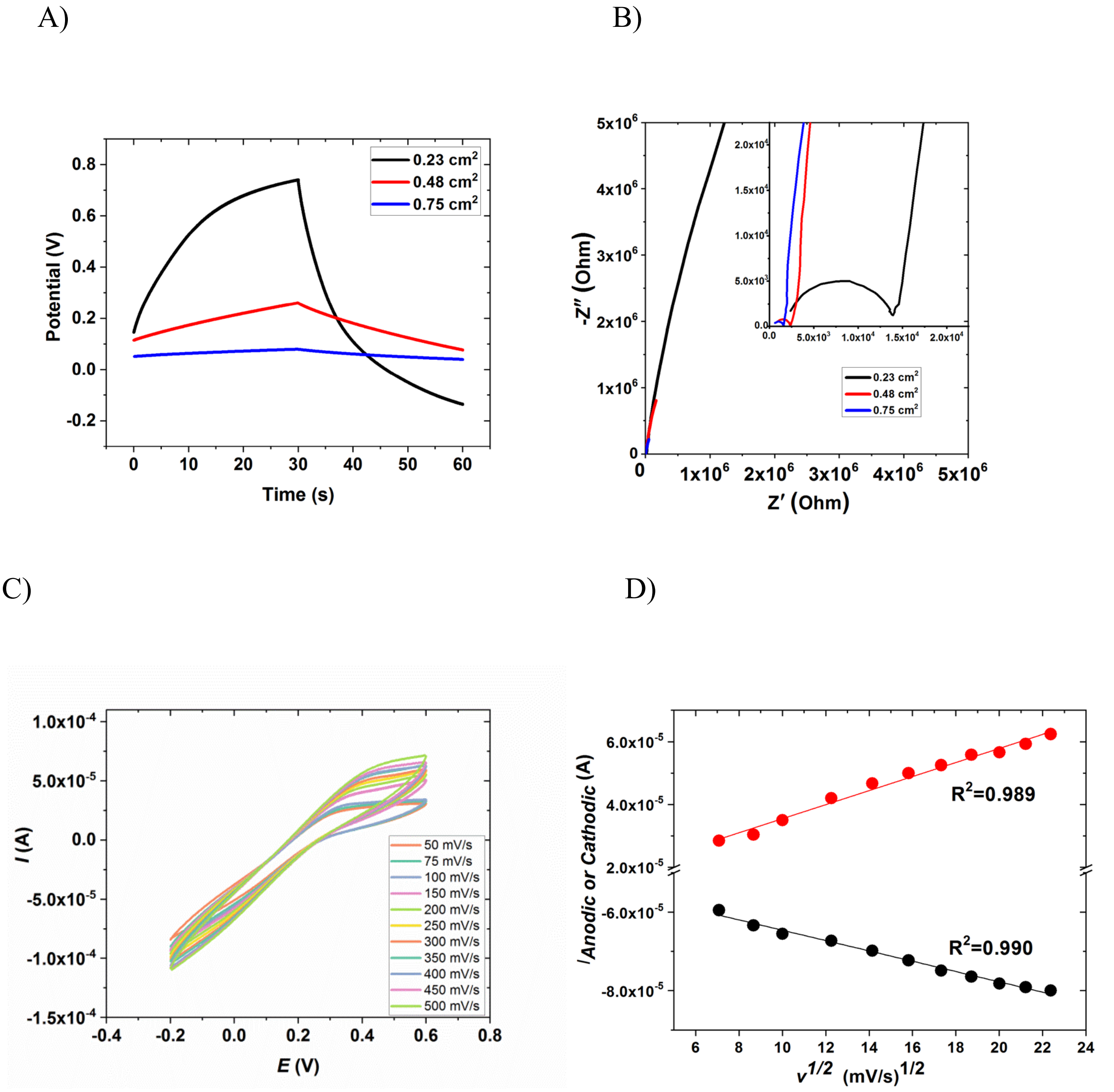

Figure S3 A) Chronopotentiometric curves (I = 1* 10^{-7} A) recorded in in 0.1 M KCl and B) EIS spectra recorded for PLA insulated CB-PLA electrodes in 0.1 M KCl at 0.3 V using amplitude 50 mV, frequency range 0.01−100000 Hz, surface areas of electrodes used: 0.23 cm^2 (black), 0.48 cm^2 (red), 0.75 cm^2 (blue). C) Current-voltage characteristics for different scan rate (50 - 500 mV/s) and D) dependence of square root of scan rate on the anodic or cathodic current recorded for PLA electrode immersed in 10 mM $K_3Fe(CN)_6$ in 0.1 M KCl, C) and D) results are obtained for disc shape electrode of 0.6 cm diameter.

A) B)

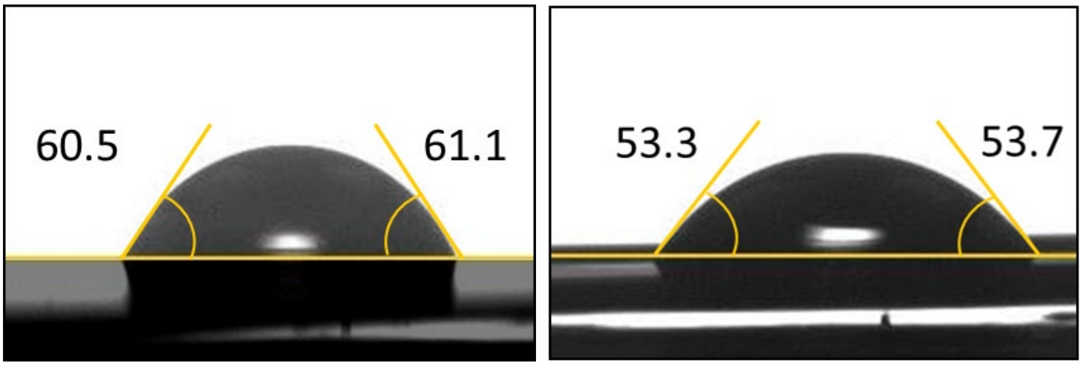

Figure S4. Contact angle of A) PLA (non-conductive) and B) CB-PLA (conductive).

A) B)

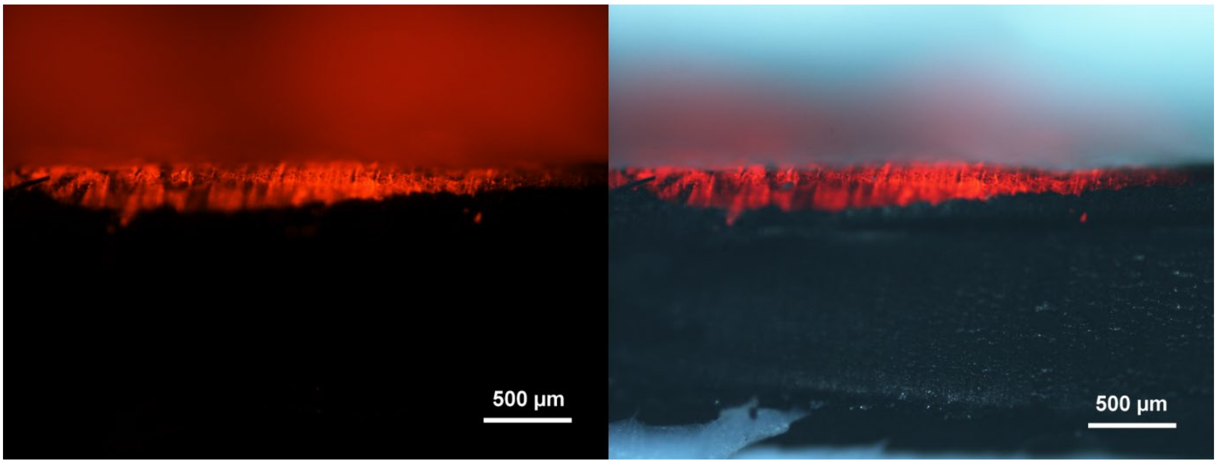

Figure S5. Fluorescence microscopy image (A), and merged UV/VIS image with fluorescence microscopy images (B) of: the cross section of CB-PLA surface top modified with 3 µL drop of NR solution in THF (magnification 5 times), the image was recorded after evaporation of THF.

A) B)

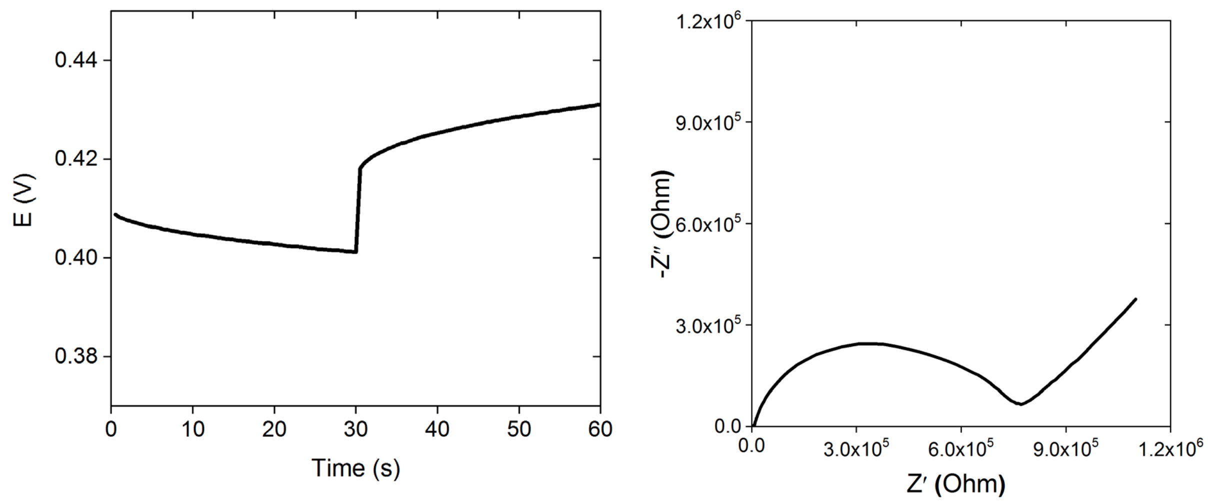

Figure S6. Electrochemical characteristics of 3D-K^+-ISE: A) chronopotentiometric dependences I = 10^{-8}A, cathodic polarization followed by anodic polarization B) EIS spectrum recorded in 0.1 M KCl at 0.3 V using amplitude 50 mV, frequency range 0.01−100000 Hz.

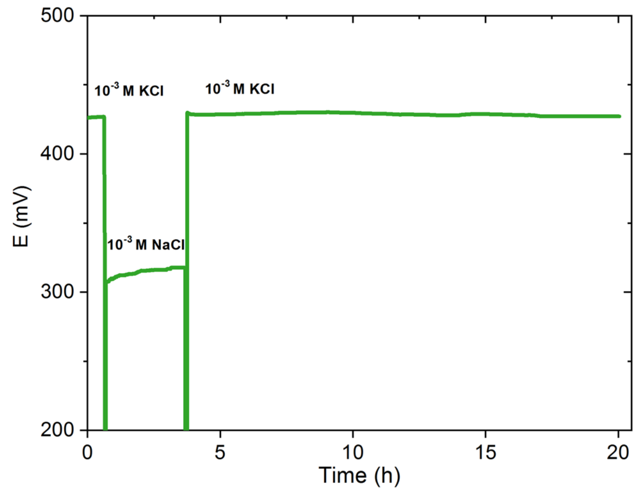

Figure S7. Water layer test of 3D-K^+-ISE support performed in 10^{-3} M primary ion (K^+) and interfering ion (Na^+) solutions.

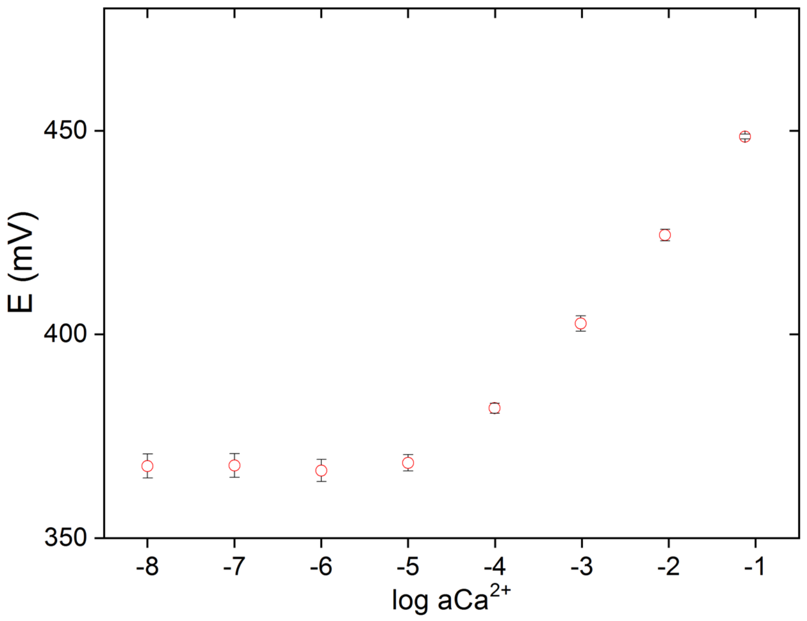

Figure S8. Within day stability of 3D-Ca^{2+}-ISE.

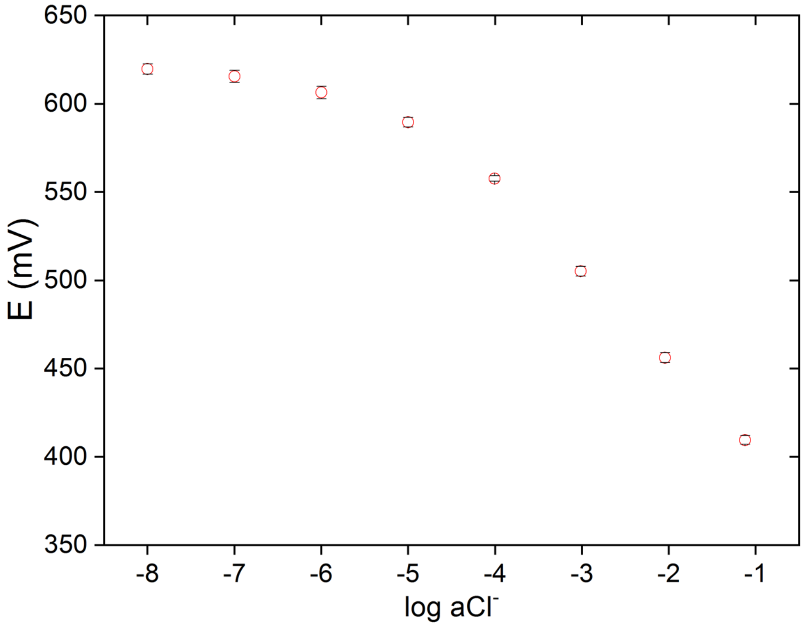

Figure S9. Within day stability of 3D-Cl^{-}-ISE.
